# Supplementary material for: Cellular mechano-environment regulates the mammary circadian clock
Source: Nat Commun. 2017 Jan 30;8:14287. doi: 10.1038/ncomms14287 (PMC5290282; doi:10.1038/ncomms14287)
Supplement: Supplementary Information — Supplementary Figures and Supplementary Tables. [file ncomms14287-s1.pdf]

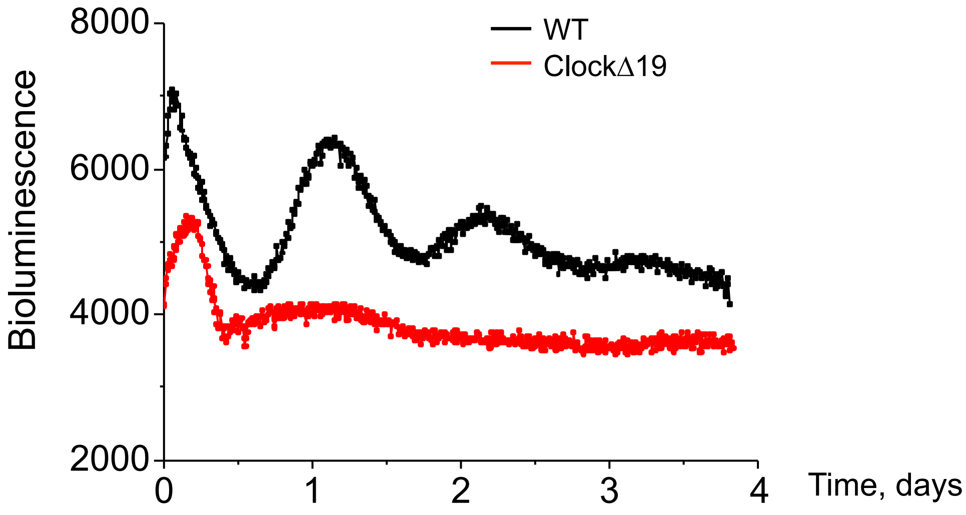

Supplementary Fig. 1. Loss of PER2::Luc rhythm in mammary tissue explants from ClockΔ19 mouse.

Representative PER2::Luc tissue explants isolated from 10 weeks old WT (black) or ClockΔ19 mouse (red) were recorded using photon counting tubes. N=4 animals.

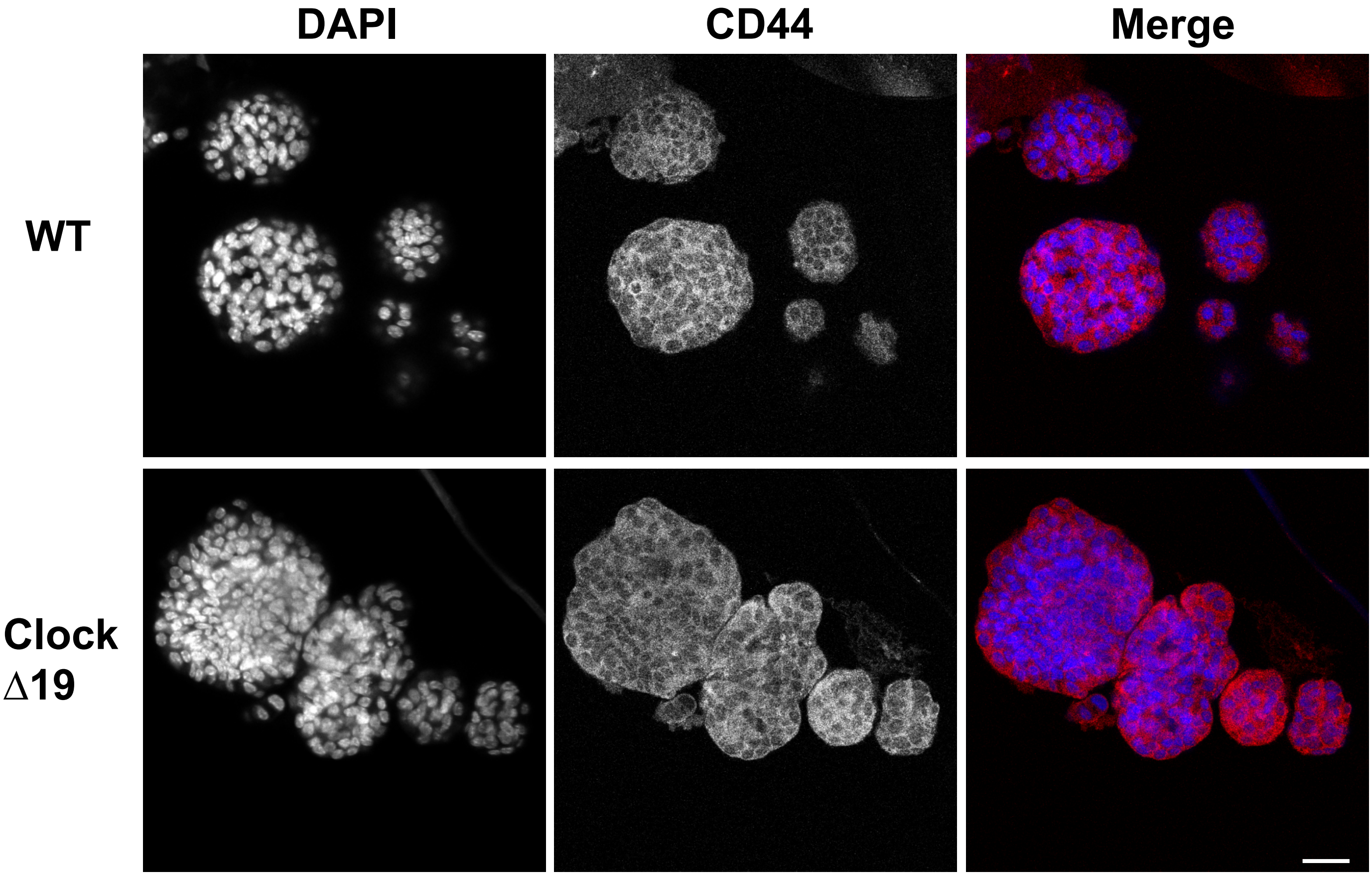

Supplementary Fig. 2. CD44 staining in mammospheres prepared from WT or ClockΔ19 mice.

Primary mammosphere assay was performed at a cell density of 2,000 cells/cm^2 from wild-type or ClockΔ19 mutant mice. Note that very few cell clusters were identified in the ClockΔ19 cultures, but a few clusters were present in comparison to multiple clusters at this cell seeding concentration from WT. N=3 animals, scale bar = 50 μm. Red, CD44; blue, DAPI.

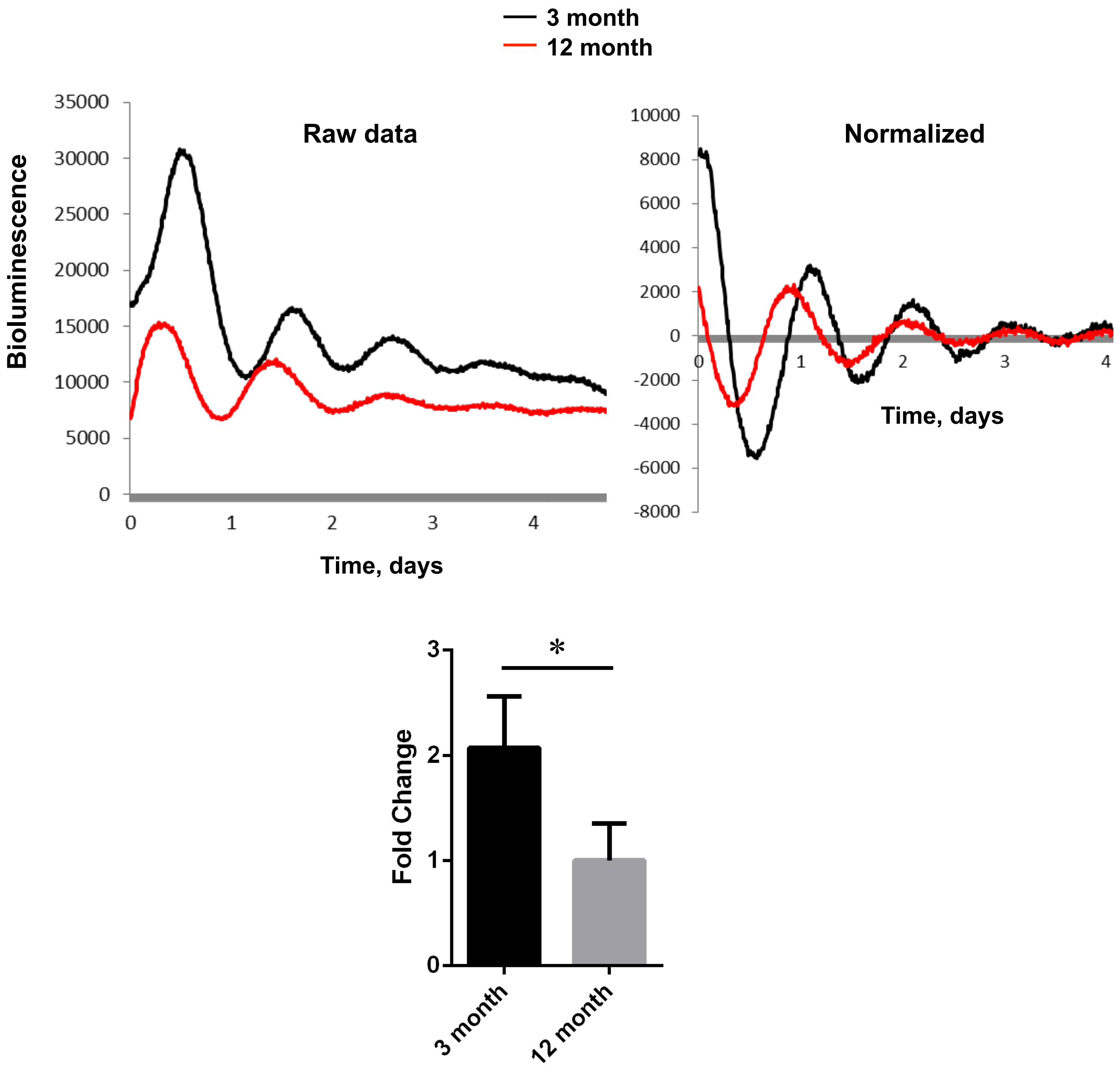

Supplementary Fig. 3. Age-related decline of circadian amplitude in mammary tissue.
Top: representative PER2::Luc traces from mammary glands of either 3 month (black) or 12 month old (red) female mice, both raw data and normalised data are shown. Bottom: fold change of amplitude was quantified. Student's t-test, mean ± SEM, *$p<0.05$, n=3 animals.

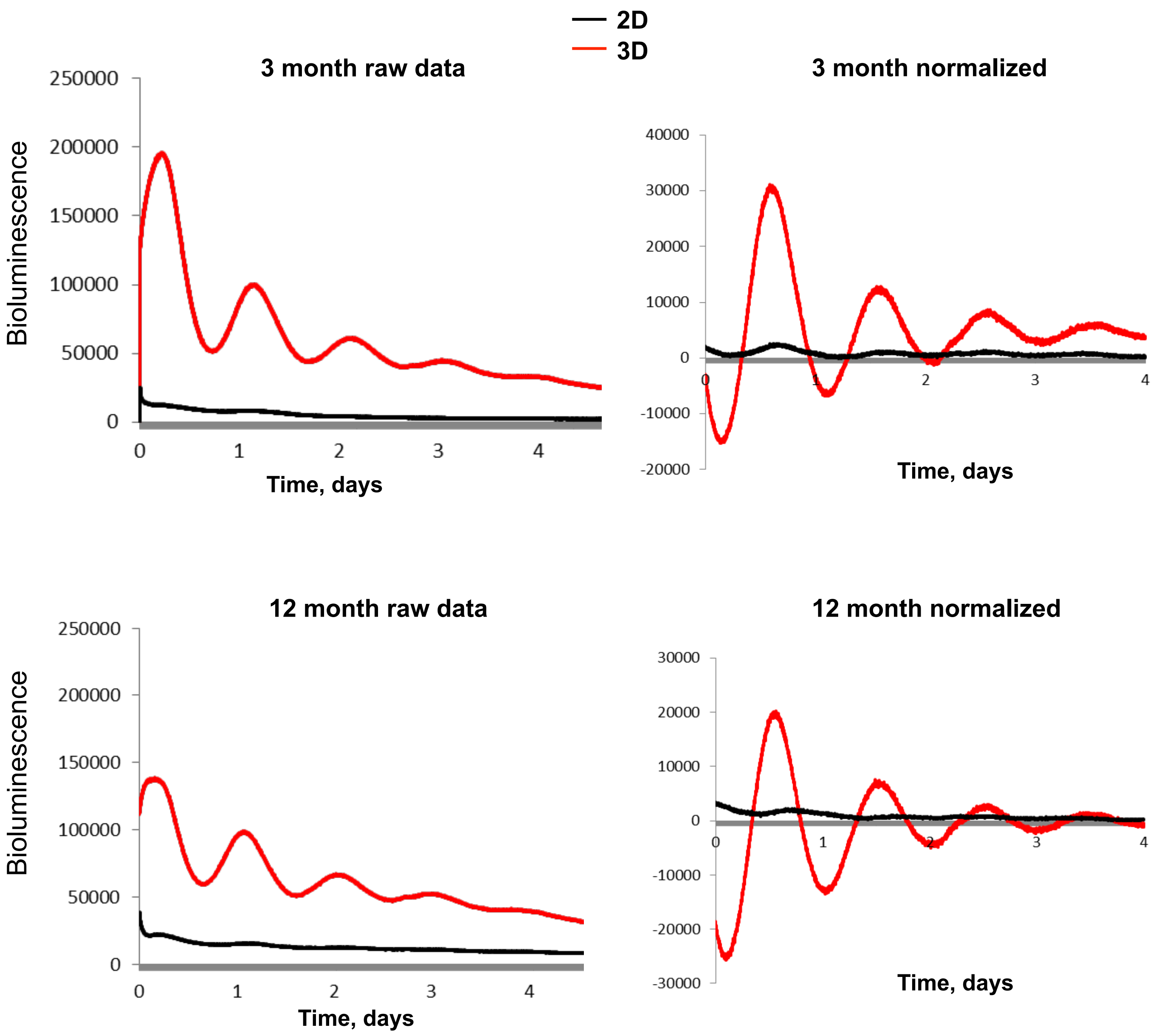

Supplementary Fig. 4. Circadian oscillations from MECs isolated from young and older mice.

Primary MECs from 3 month and 12 month old mice were seeded into 35mm dishes coated with either collagen (2D, black) or Matrigel (3D, red). Representative bioluminescence recording results were shown. Note that in both age groups, cells in 3D have robust oscillations, while cells in 2D have disrupted circadian rhythms. Student's t-test, mean ± SEM, $^*p < 0.05$, n=3 animals.

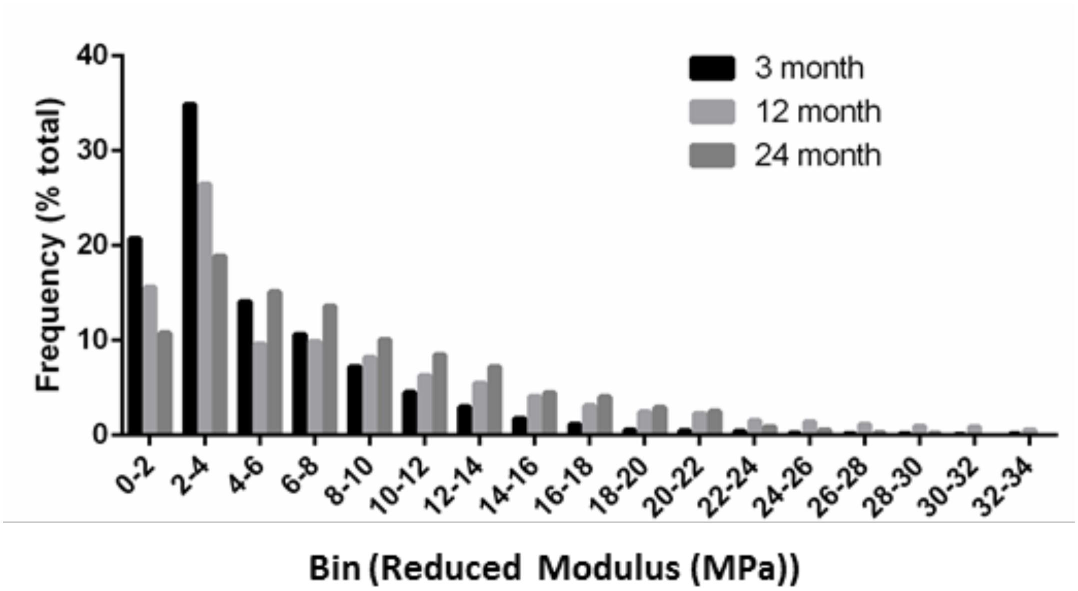

Supplementary Fig. 5. Frequency distribution for mammary gland AFM data.
Y-axis represents the frequency of counts of data points that fell within a particular bin as a percentage of total number of values. A right shifted distribution of the reduced modulus in the older mammary gland tissues indicates increased stiffness. N=6 animals, 1200 replicates.

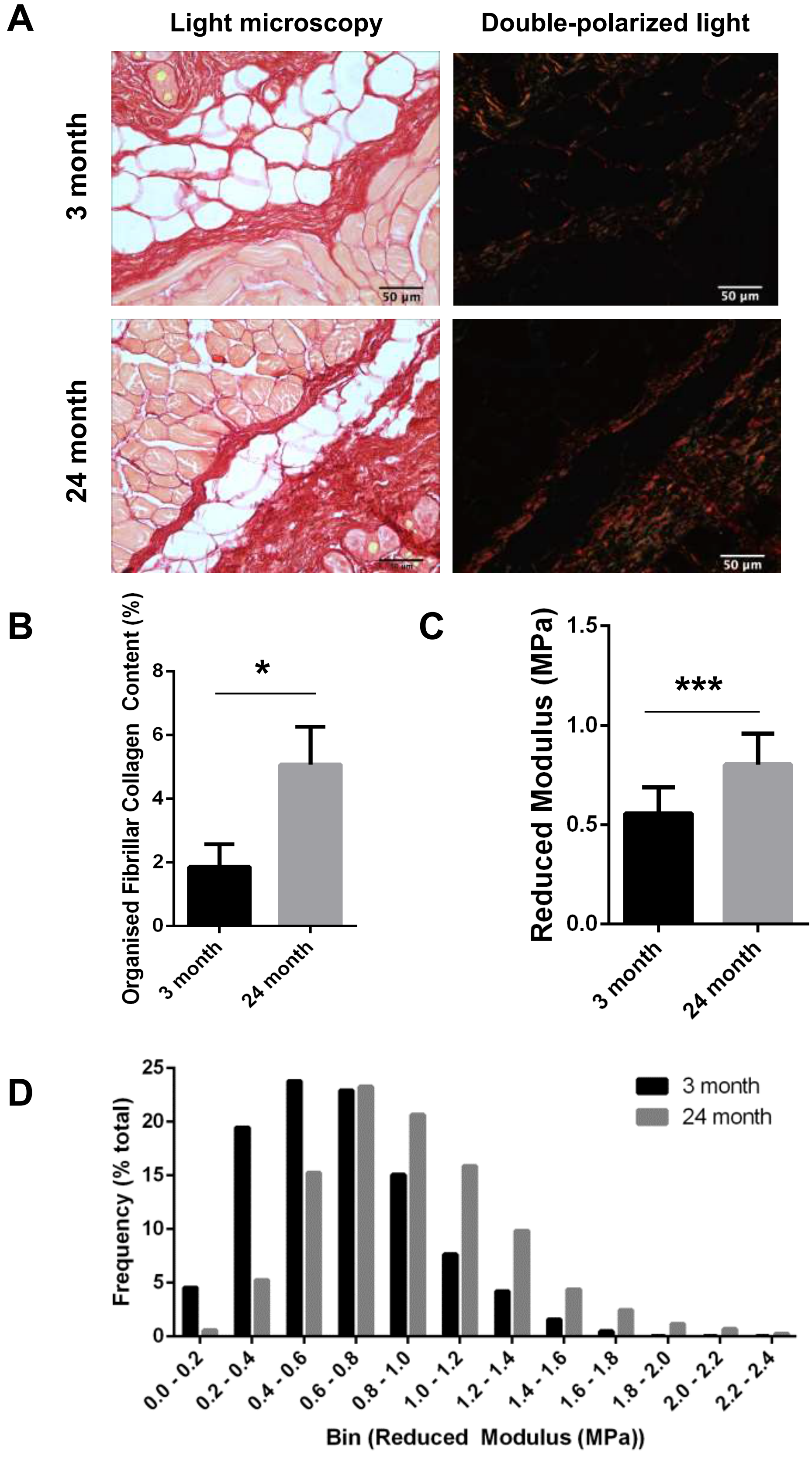

Supplementary Fig. 6. Picrosirius red and AFM measurements in young and old skin tissues.
(A) Picrosirius red staining under brightfield (left panel) and double-polarized light (right panel) of 3 month and 24 month old skin sections. (B) The % of organized fibrillar collagen was quantified. Student's t-test, mean ± SEM, $*p < 0.05$, n=3 animals. (C) AFM measurement of tissue stiffness. Mann-Whitney U Test, mean ± SEM, $***p < 0.001$, n=3 animals, 300 replicates. (D) Frequency distribution for skin AFM data. A right shifted distribution of the reduced modulus in the skin tissues, indicating increased stiffness.

A

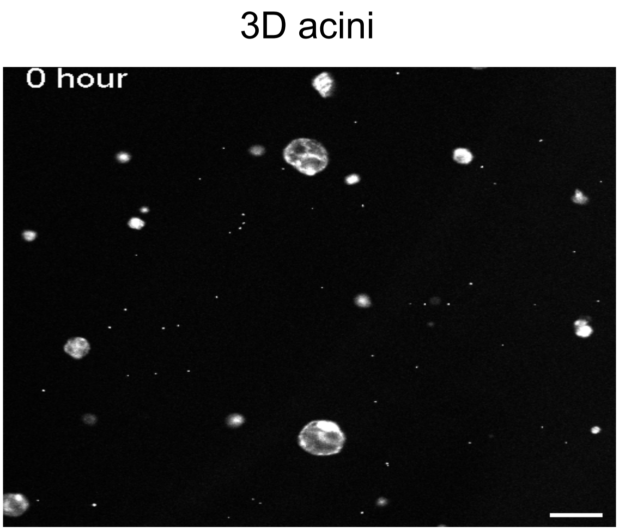

B

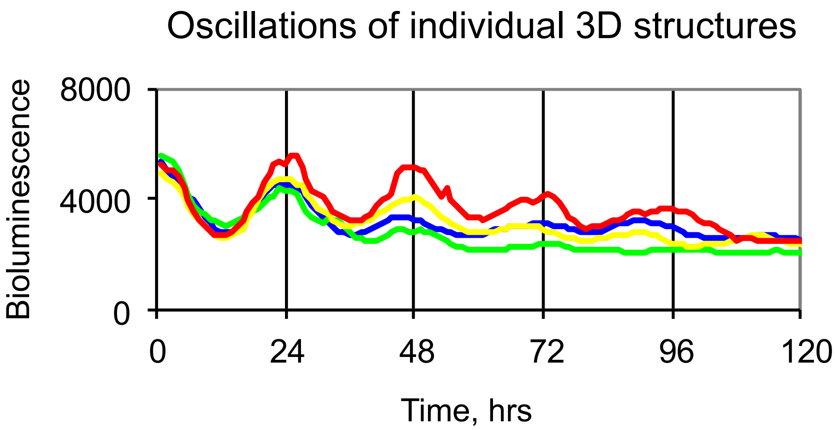

Supplementary Fig. 7. Real-time bioluminescence imaging of primary 3D PER2::Luc acini. (A) Bioluminescence imaging of primary MEC cells cultured in 3D. Scale bar = 100 μm. (B) Quantification of real-time bioluminescence signals from individual acini structures. Four representative traces were shown. N=3 animals.

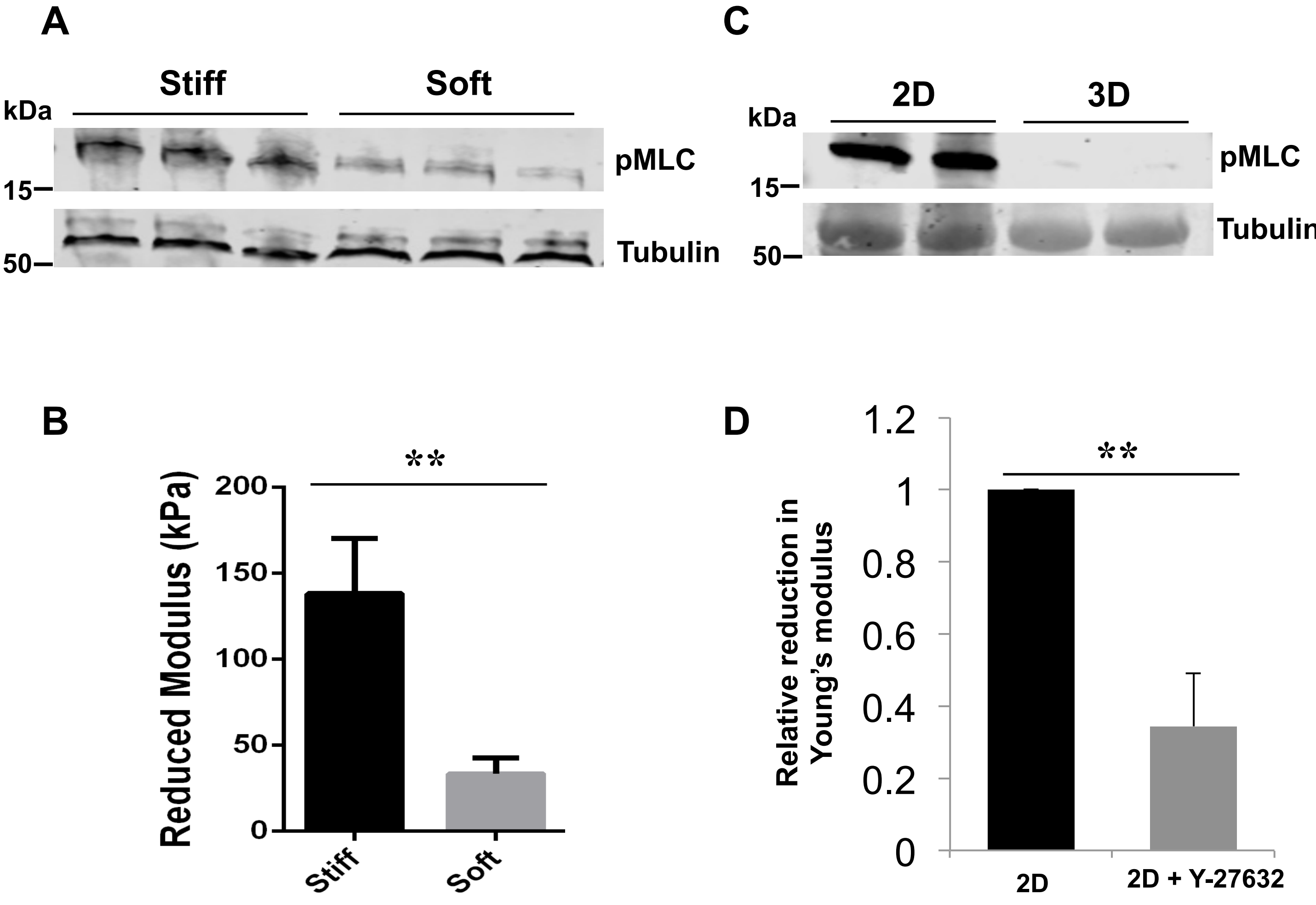

Supplementary Fig. 8. Altered actin cytoskeleton in MECs cultured under different mechano-environmental conditions.

(A) Immuno-blot of pMLC in cells cultured at different stiffness using the 3D alginate gel system. N=3 animals. (B) AFM quantification of the stiff and soft 3D alginate matrix, Mann-Whitney U Test, mean ± SEM, **p < 0.01, n=3 animals, 300 replicates. (C) Western blot of pMLC expression in cells cultured on either 2D or 3D. N=3 animals. (D) AFM quantification of MEC cells cultured on 2D collagen, treated with Y-27632 (30 μM). Student's t-test, mean ± SEM, **p < 0.01, n=3 replicates.

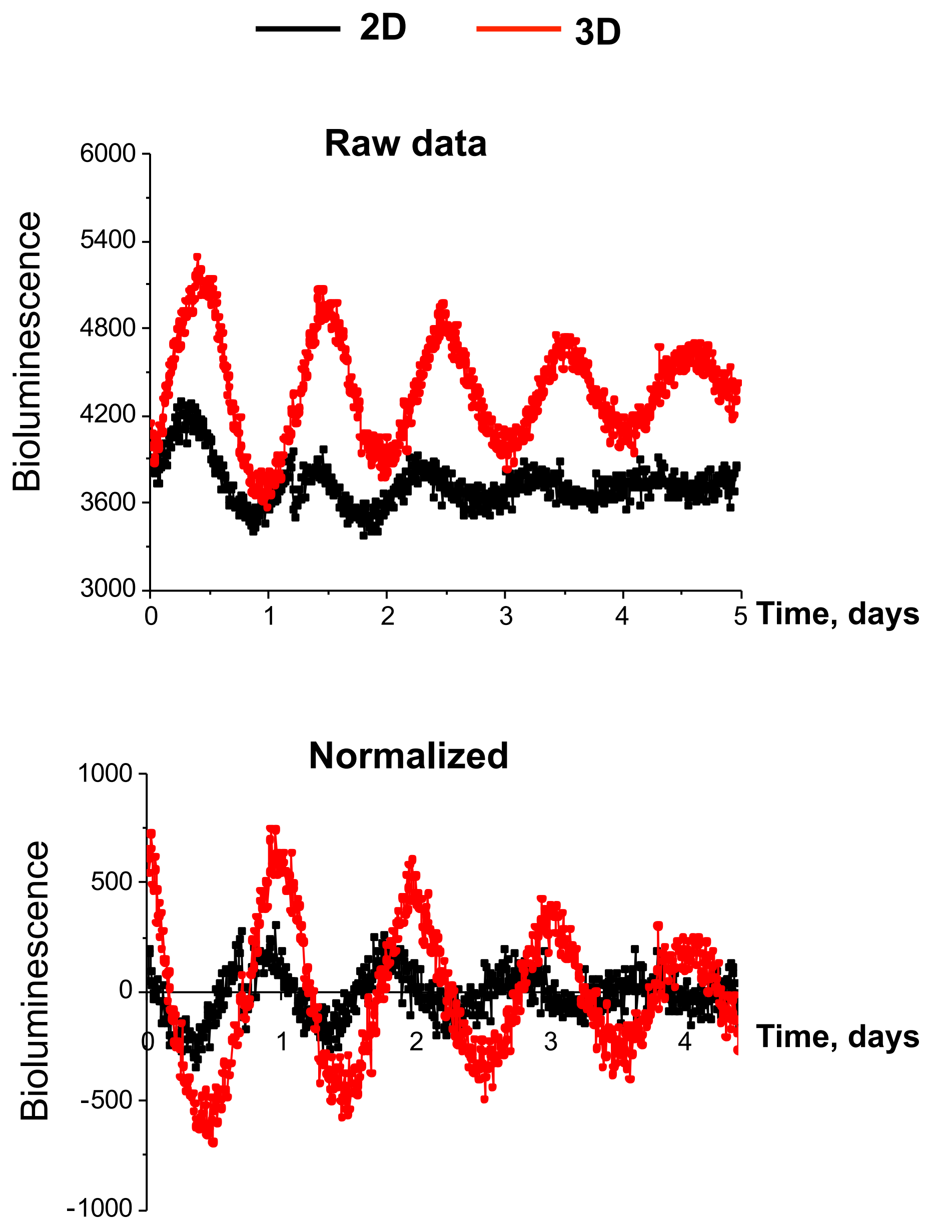

Supplementary Fig. 9. The circadian clock in lung epithelial cells is regulated by its microenvironment.

Primary lung epithelia were isolated from the PER2::Luc mice and cultured on either 2D (black) or 3D (red) ECM. Real-time bioluminescence recording of the same number of cells revealed >2 fold suppression of lung epithelial clock in 2D condition, n=4 animals. Top: raw data. Bottom: normalized data.

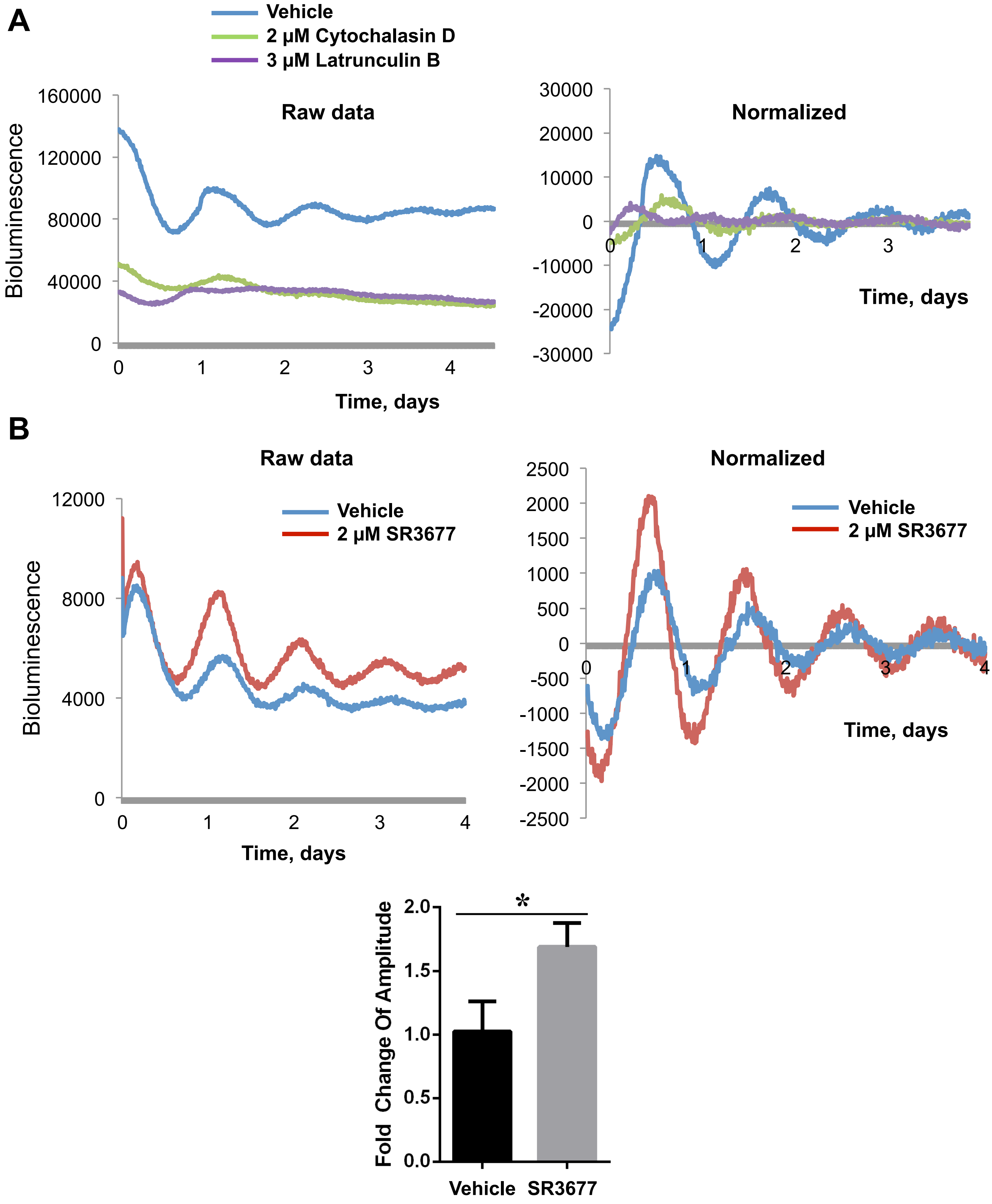

Supplementary Fig. 10. Effect of SR3677, cytochalasin C and Latrunculin B on the MEC circadian clock.

(A) Representative bioluminescence traces of PER2::Luc MECs cultured in 3D Matrigel, treated with Cytochalasin D (green) and Latrunculin B (purple). Note the severely disrupted circadian rhythm by both compounds. Control treatment is in blue. N=3 animals. (B) Representative bioluminescence traces (top) and quantification (bottom) of fold change of PER2::Luc amplitude (based on first peak) of primary MECs cultured on 2D collagen, treated with vehicle (blue) or SR3677 ROCK inhibitor (red). Student's t-test, mean ± SEM, *$p < 0.05$, n=4 animals.

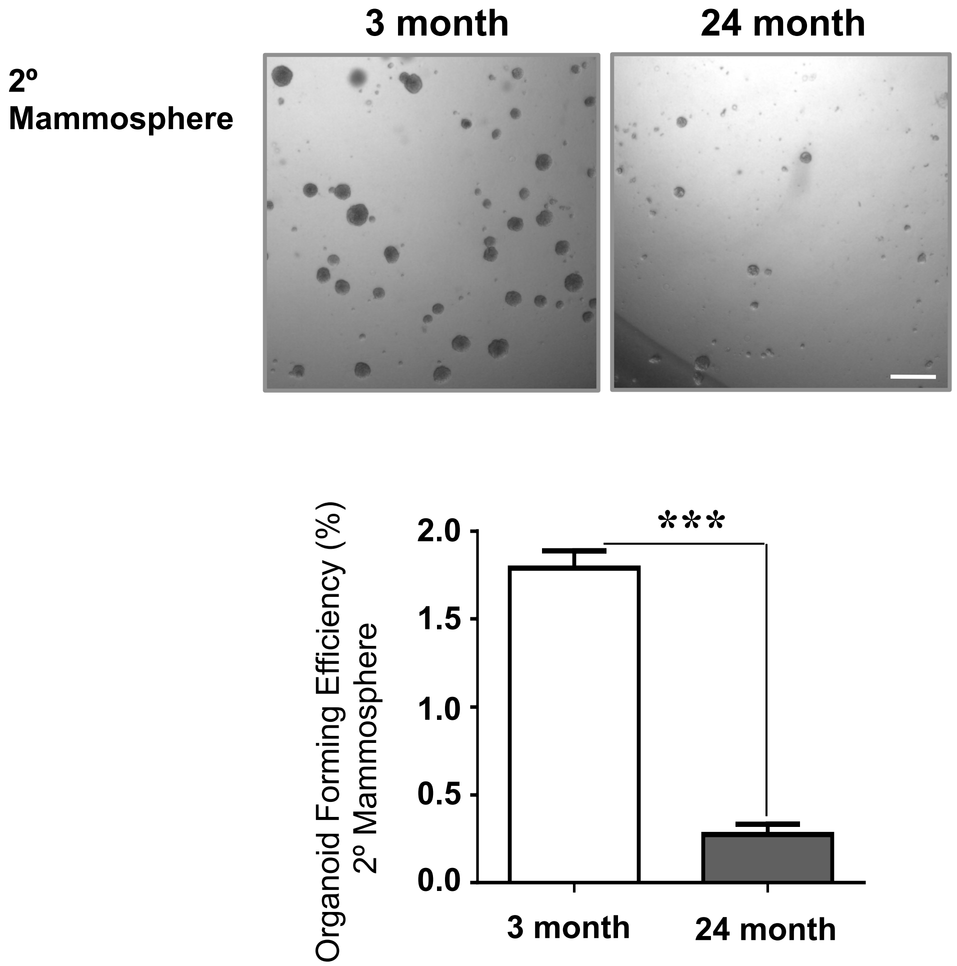

Supplementary Fig. 11. Self-renewal of mammary stem cells is compromised by ageing. Primary MECs from 3 or 24 month old WT mice were plated as single cells in mammosphere cultures, then cell clusters were dissociated and similar numbers of single cells were plated for secondary mammosphere assays for 10 days. N=3 animals, Scale bar = 100 µm. Quantification of the mammosphere-forming efficiency is shown on the bottom. Student's t-test, data are shown as mean ± SEM ***$p < 0.001$.

Fig 7B

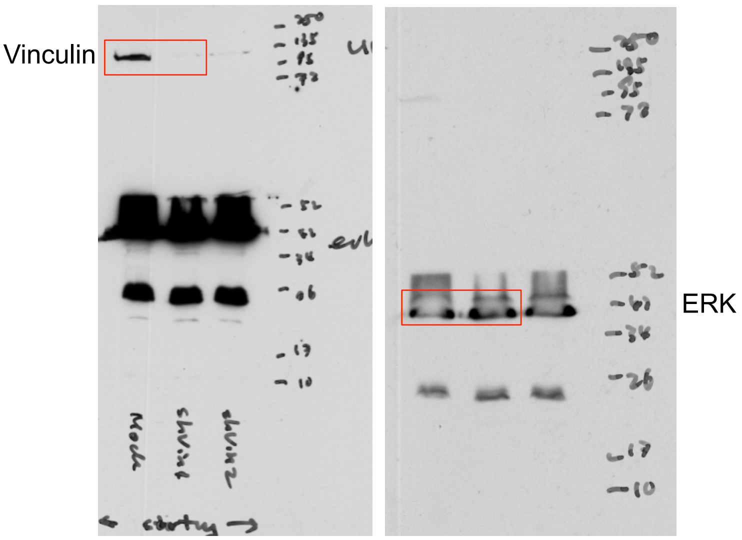

Fig 8A

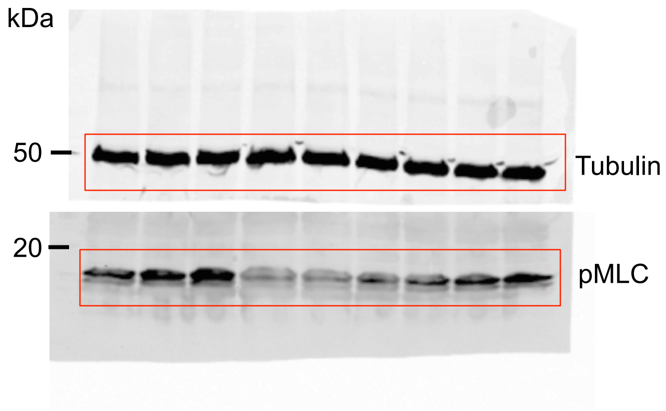

Fig 9E

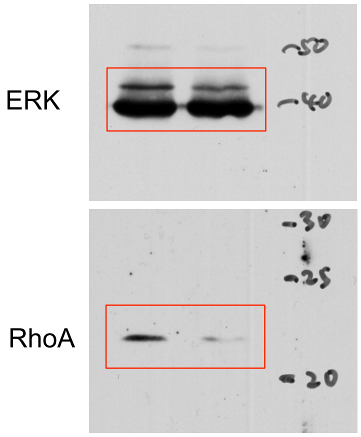

Supplementary Fig 8A

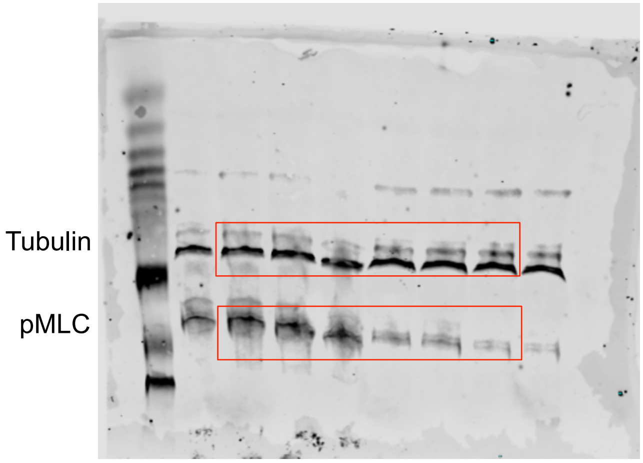

Supplementary Fig 8C

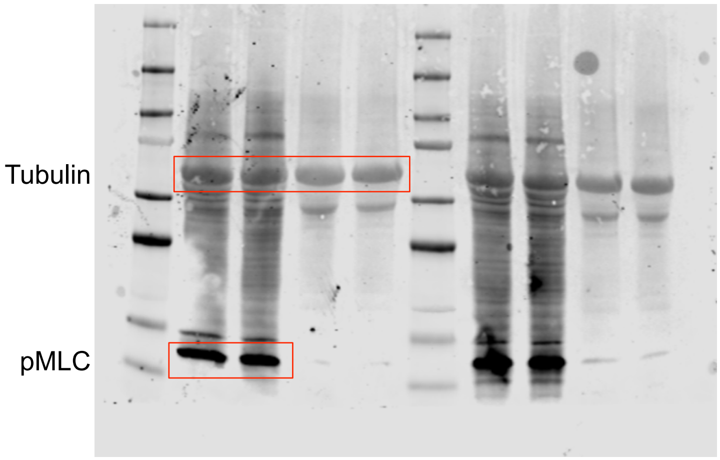

Supplementary Fig. 12. Uncropped scans of the western blots shown in paper (boxed bands). Data correspond to Figs 7B, 8A, 9E and Supplementary Figs 8A, 8C.

Supplemetary Table 1: List of genes with circadian expression (~24 hour period) in mouse mammary gland.
Ranking is based on their goodness of fit to a 24hr sine-wave model (Rsq-value, > 0.63 as a cut-off)

Name	Gene Title	JTK ADJ.P	Circwave Rsq1	Circwave q1
Nr1d2	nuclear receptor subfamily 1, group D, member 2	9.57E-05	0.9779	0
Per3	Period homolog 3 (Drosophila)	2.64E-05	0.9707	0
Usp2	ubiquitin specific peptidase 2	0.000294162	0.9585	0
Por	P450 (cytochrome) oxidoreductase	0.000794327	0.9507	0
Hlf	hepatic leukemia factor	9.57E-05	0.9481	0
Crtac1	cartilage acidic protein 1	0.001933622	0.9475	0
Nampt	nicotinamide phosphoribosyltransferase	9.57E-05	0.9461	0
Tmem55b	transmembrane protein 55b	0.000294162	0.9459	0
Rsad2	radical S-adenosyl methionine domain containing 2	0.001933622	0.9416	0
Hist1h1c	histone cluster 1, H1c	2.64E-05	0.9374	0
Abcg2	ATP-binding cassette, sub-family G (WHITE), member 2	0.000294162	0.9365	0
Dnajb4	DnaJ (Hsp40) homolog, subfamily B, member 4	0.004320437	0.9359	0
Slc22a3	solute carrier family 22 (organic cation transporter), member 3	2.64E-05	0.931	0
Tmem48	transmembrane protein 48	0.000794327	0.9306	0
Rgs7bp	regulator of G-protein signalling 7 binding protein	0.000794327	0.9304	0
Cln3	ceroid lipofuscinosis, neuronal 3, juvenile (Batten, Spielmeyer-Vogt disease)	0.001933622	0.9298	0
Lhfpl2	lipoma HMGIC fusion partner-like 2	0.000794327	0.9288	0
2810026P18R	RIKEN cDNA 2810026P18 gene	0.000294162	0.9286	0
C730049O14R	RIKEN cDNA C730049O14 gene	0.001933622	0.9266	0
Cys1	cystin 1	9.57E-05	0.9256	0
Acsm5	acyl-CoA synthetase medium-chain family member 5	0.008979903	0.9242	0
Map2k3	mitogen-activated protein kinase kinase 3	9.57E-05	0.9242	0
N4bp3	NEDD4 binding protein 3	0.008979903	0.924	0
Dab2ip	disabled homolog 2 (Drosophila) interacting protein	0.001933622	0.9234	0
Efr3a	EFR3 homolog A (S. cerevisiae)	0.001933622	0.9205	0
Tef	thyrotroph embryonic factor	9.57E-05	0.9193	0
Arntl	aryl hydrocarbon receptor nuclear translocator-like	0.001933622	0.918	0
Sox17	SRY-box containing gene 17	0.001933622	0.9168	0
Cln5	ceroid-lipofuscinosis, neuronal 5	0.008979903	0.9153	0
Sybu	syntabulin (syntaxin-interacting)	5.79E-06	0.9146	0
Eef2k	eukaryotic elongation factor-2 kinase	5.79E-06	0.9138	0
Cry1	cryptochrome 1 (photolyase-like)	0.001933622	0.9138	0
Fgfrl1	fibroblast growth factor receptor-like 1	0.004320437	0.9136	0
Tollip	toll interacting protein	0.001363975	0.9131	0
Dem1	defects in morphology 1 homolog (S. cerevisiae)	0.000294162	0.9128	0
Timp3	tissue inhibitor of metalloproteinase 3	0.000794327	0.9121	0
Rgs7bp	regulator of G-protein signalling 7 binding protein	0.001933622	0.912	0
Atxn7l3b	ataxin 7-like 3B	5.79E-06	0.9117	0
Slc1a5	solute carrier family 1 (neutral amino acid transporter), member 5	9.57E-05	0.9109	0
Gab2	growth factor receptor bound protein 2-associated protein 2	0.000794327	0.9107	0
Sec14l1	SEC14-like 1 (S. cerevisiae)	0.000794327	0.9103	0
Fam122b	family with sequence similarity 122, member B	0.008979903	0.9073	0
Fmo5	flavin containing monooxygenase 5	0.004320437	0.9071	0
Rnf125	ring finger protein 125	0.000794327	0.905	0
Itga6	integrin alpha 6	0.001933622	0.9045	0
Cp	ceruloplasmin	0.008979903	0.9029	0
Sec24b	Sec24 related gene family, member B (S. cerevisiae)	0.003127029	0.9029	0
Alas1	aminolevulinic acid synthase 1	0.001933622	0.9025	0
Irak1bp1	interleukin-1 receptor-associated kinase 1 binding protein 1	0.000794327	0.9018	0
Cldn5	claudin 5	0.001933622	0.9005	0
Nfx1	nuclear transcription factor, X-box binding 1	0.004320437	0.8995	0
Cdkn1a	cyclin-dependent kinase inhibitor 1A (P21)	0.000794327	0.8991	0
Cmklr1	chemokine-like receptor 1	0.001933622	0.899	0
Asns	asparagine synthetase	0.001933622	0.899	0
Npy1r	neuropeptide Y receptor Y1	0.000294162	0.8978	0
Tmem57	transmembrane protein 57	0.001933622	0.8965	0
Pdxk	pyridoxal (pyridoxine, vitamin B6) kinase	0.000794327	0.8962	0
Tbl2	transducin (beta)-like 2	0.004320437	0.8945	0
Srm	spermidine synthase	0.004320437	0.8922	0
Slc25a36	Solute carrier family 25, member 36	0.008979903	0.892	0
Ipo5	importin 5	5.79E-06	0.8908	0
Glrx	glutaredoxin	0.004320437	0.8906	0
Abcd2	ATP-binding cassette, sub-family D (ALD), member 2	0.008979903	0.8898	0
Tpd52l2	tumor protein D52-like 2	0.000794327	0.888	0.000984364
Smad6	MAD homolog 6 (Drosophila)	0.000794327	0.8878	0.000984364
Dnajb6	DnaJ (Hsp40) homolog, subfamily B, member 6	9.57E-05	0.8873	0.000984364
Nadk	NAD kinase	0.000794327	0.8859	0.000984364
Gmcl1	germ cell-less homolog 1 (Drosophila)	0.000794327	0.8858	0.000984364
Fmo2	flavin containing monooxygenase 2	0.004320437	0.883	0.000984364
Mocos	molybdenum cofactor sulfurase	0.001933622	0.8818	0.000984364
Myd88	myeloid differentiation primary response gene 88	0.000794327	0.8813	0.000984364
Tmem35	transmembrane protein 35	0.008979903	0.8797	0.000984364
Ypel1	yippee-like 1 (Drosophila)	0.004320437	0.8793	0.000984364
Stx12	syntaxin 12	0.008979903	0.8778	0.000984364
AI429363	expressed sequence AI429363	0.004320437	0.8775	0.000984364
Gpr146	G protein-coupled receptor 146	0.008979903	0.8772	0.000984364

Ctsh	cathepsin H	0.000794327	0.8764	0.000984364
P4ha1	procollagen-proline, 2-oxoglutarate 4-dioxygenase (proline 4-hydroxylase), alpha 1 polyp	0.004320437	0.8761	0.000984364
Selenbp1	selenium binding protein 1	0.004320437	0.8759	0.000984364
Arhgap20	Rho GTPase activating protein 20	0.000294162	0.8755	0.000984364
Ramp2	receptor (calcitonin) activity modifying protein 2	0.001933622	0.8754	0.000984364
Vps13a	vacuolar protein sorting 13A (yeast)	0.001933622	0.8752	0.000984364
Ankle2	ankyrin repeat and LEM domain containing 2	0.008979903	0.8752	0.000984364
Atxn10	ataxin 10	0.001933622	0.8752	0.000984364
Cidec	cell death-inducing DFFA-like effector c	0.000294162	0.8743	0.000984364
Dennd2a	DENN/MADD domain containing 2A	0.004320437	0.8733	0.000984364
Hipk1	homeodomain interacting protein kinase 1	0.001933622	0.8732	0.000984364
Dapk1	death associated protein kinase 1	0.000794327	0.8729	0.000984364
Hsd17b7	hydroxysteroid (17-beta) dehydrogenase 7	0.001933622	0.8707	0.000984364
Ankrd50	ankyrin repeat domain 50	0.004320437	0.8705	0.000984364
Kctd15	potassium channel tetramerisation domain containing 15	0.004320437	0.8695	0.000984364
Cyp2e1	cytochrome P450, family 2, subfamily e, polypeptide 1	0.001933622	0.8692	0.000984364
Ccdc85b	coiled-coil domain containing 85B	0.008979903	0.8689	0.000984364
Galnt2	UDP-N-acetyl-alpha-D-galactosamine:polypeptide N-acetylgalactosaminyltransferase 2	0.004320437	0.8688	0.000984364
Fsd2	fibronectin type III and SPRY domain containing 2	0.001933622	0.8686	0.000984364
Cars2	cysteinyl-tRNA synthetase 2 (mitochondrial)(putative)	0.000794327	0.8677	0.000984364
2810405F17Ri	RIKEN cDNA 2810405F17 gene	0.004320437	0.8673	0.000984364
Fbxo45	F-box protein 45	0.000794327	0.8667	0.000984364
Dnaja2	DnaJ (Hsp40) homolog, subfamily A, member 2	0.008979903	0.8666	0.000984364
Apob48r	apolipoprotein B48 receptor	0.008979903	0.8664	0.000984364
B230120H23R	RIKEN cDNA B230120H23 gene	0.008979903	0.8655	0.000984364
Mar-05	membrane-associated ring finger (C3HC4) 5	0.001933622	0.8636	0.000984364
Isca2	iron-sulfur cluster assembly 2 homolog (S. cerevisiae)	0.008979903	0.8631	0.000984364
Plagl1	pleiomorphic adenoma gene-like 1	0.000294162	0.8628	0.000984364
Herpud1	homocysteine-inducible, endoplasmic reticulum stress-inducible, ubiquitin-like domain m	0.001933622	0.8627	0.000984364
Cgrrf1	cell growth regulator with ring finger domain 1	0.000794327	0.8616	0.000984364
Rgs2	regulator of G-protein signaling 2	0.004320437	0.8589	0.000984364
Lca5l	Leber congenital amaurosis 5-like	0.008979903	0.8584	0.001349128
Zfp207	zinc finger protein 207	0.008979903	0.8581	0.001349128
Riok2	RIO kinase 2 (yeast)	0.004320437	0.8575	0.001349128
Fut8	fucosyltransferase 8	0.004320437	0.857	0.001349128
Ncaph	non-SMC condensin I complex, subunit H	0.001933622	0.857	0.001349128
Tmx1	thioredoxin-related transmembrane protein 1	0.008979903	0.8568	0.001349128
Dram1	DNA-damage regulated autophagy modulator 1	0.000794327	0.8556	0.001349128
H6pd	hexose-6-phosphate dehydrogenase (glucose 1-dehydrogenase)	0.001933622	0.8552	0.001349128
Ppm1a	protein phosphatase 1A, magnesium dependent, alpha isoform	0.008979903	0.8547	0.001349128
2310009A05R	RIKEN cDNA 2310009A05 gene	9.57E-05	0.8536	0.001349128
AB099516 ///	cDNA sequence AB099516 /// predicted gene 10035 /// HIG1 domain family, member 1C	0.000794327	0.8526	0.001349128
LOC10050367	60S ribosomal protein L5-like	0.000794327	0.8523	0.001349128
Pard6g	par-6 partitioning defective 6 homolog gamma (C. elegans)	0.001933622	0.8523	0.001349128
Smoc1	SPARC related modular calcium binding 1	0.008979903	0.8522	0.001349128
Smtnl2	smoothelin-like 2	9.57E-05	0.8515	0.001349128
Etv6	ets variant gene 6 (TEL oncogene)	0.000794327	0.8515	0.001349128
Gm11627	predicted gene 11627	0.004320437	0.851	0.001349128
Nup88	nucleoporin 88	0.008979903	0.8509	0.001349128
Adora1	adenosine A1 receptor	0.008979903	0.8503	0.001349128
Arhgap29	Rho GTPase activating protein 29	0.001933622	0.8497	0.001349128
Pigc	phosphatidylinositol glycan anchor biosynthesis, class C	0.000794327	0.8492	0.001349128
Zfp330	zinc finger protein 330	0.004320437	0.8489	0.001349128
Dtx2	deltex 2 homolog (Drosophila)	0.001933622	0.8484	0.001349128
Phlda3	pleckstrin homology-like domain, family A, member 3	0.000794327	0.8479	0.001349128
Ints8	integrator complex subunit 8	0.008979903	0.8468	0.001349128
Hnrnpk	heterogeneous nuclear ribonucleoprotein K	0.008979903	0.8457	0.001349128
Ttc39c	tetratricopeptide repeat domain 39C	0.000794327	0.8456	0.001349128
Golt1b	golgi transport 1 homolog B (S. cerevisiae)	0.008979903	0.8454	0.001349128
Dctd	dCMP deaminase	0.004320437	0.8448	0.001349128
Prkce	protein kinase C, epsilon	0.000294162	0.8447	0.001349128
Zhx3	zinc fingers and homeoboxes 3	0.001933622	0.8445	0.001349128
Eif2c3	eukaryotic translation initiation factor 2C, 3	0.004320437	0.844	0.001349128
Cnksr3	Cnksr family member 3	0.008979903	0.8432	0.001349128
Rnf121	ring finger protein 121	0.004320437	0.8425	0.001349128
Trim37	tripartite motif-containing 37	0.004320437	0.8424	0.001349128
Cdc40	cell division cycle 40 homolog (yeast)	0.001933622	0.8423	0.001349128
Ahcyl2	S-adenosylhomocysteine hydrolase-like 2	9.57E-05	0.8423	0.001349128
Clec7a	C-type lectin domain family 7, member a	0.008979903	0.8423	0.001349128
D4Ertd22e	DNA segment, Chr 4, ERATO Doi 22, expressed	0.004320437	0.8418	0.001349128
Marveld1	MARVEL (membrane-associating) domain containing 1	0.000794327	0.8414	0.001640607
Gm6277	predicted gene 6277	0.004320437	0.8404	0.001640607
Nfic	nuclear factor I/C	0.001933622	0.8404	0.001640607
Rbpms	RNA binding protein gene with multiple splicing	0.000794327	0.8403	0.001640607
Adamts16	a disintegrin-like and metallopeptidase (reprolysin type) with thrombospondin type 1 mot	0.000294162	0.8385	0.001640607
Cnot8	CCR4-NOT transcription complex, subunit 8	0.001933622	0.8384	0.001640607
Ptch2	patched homolog 2	0.001933622	0.8383	0.001640607
Nkain1	Na+/K+ transporting ATPase interacting 1	0.008979903	0.8381	0.001640607
BC030336	cDNA sequence BC030336	0.001933622	0.8379	0.001640607
Procr	protein C receptor, endothelial	0.001933622	0.8366	0.001640607

Tns1	tensin 1	0.004320437	0.8363	0.001640607
Pik3r1	phosphatidylinositol 3-kinase, regulatory subunit, polypeptide 1 (p85 alpha)	0.000294162	0.8363	0.001640607
Per2	period homolog 2 (Drosophila)	0.008979903	0.836	0.001640607
Clock	circadian locomotor output cycles kaput	0.000794327	0.8356	0.001640607
Lasp1	LIM and SH3 protein 1	0.008979903	0.8355	0.001640607
0610007P14R	RIKEN cDNA 0610007P14 gene	0.004320437	0.8352	0.001640607
Bmp6	bone morphogenetic protein 6	0.008979903	0.8352	0.001640607
Ctsl	cathepsin L	0.001933622	0.835	0.001640607
Slc35d2	solute carrier family 35, member D2	9.57E-05	0.8341	0.001640607
Hip1	huntingtin interacting protein 1	0.008979903	0.834	0.001640607
Rnf8	ring finger protein 8	0.000294162	0.8334	0.001640607
Rps6kb1	ribosomal protein S6 kinase, polypeptide 1	0.001933622	0.8333	0.001640607
Tomm5	translocase of outer mitochondrial membrane 5 homolog (yeast)	0.008979903	0.8325	0.001640607
Prc1	protein regulator of cytokinesis 1	0.001933622	0.8323	0.001640607
Bnip2	BCL2/adenovirus E1B interacting protein 2	0.000794327	0.8318	0.001640607
Skap2	src family associated phosphoprotein 2	0.008979903	0.8317	0.001640607
Myo1c	myosin IC	0.004320437	0.8315	0.001640607
Nmt2	N-myristoyltransferase 2	0.004320437	0.8304	0.001640607
Fads3	fatty acid desaturase 3	0.004320437	0.8295	0.001640607
Aspscr1	alveolar soft part sarcoma chromosome region, candidate 1 (human)	0.008979903	0.8292	0.001839296
Ptcd2	pentatricopeptide repeat domain 2	0.008979903	0.8291	0.001839296
Fam20c	family with sequence similarity 20, member C	0.004320437	0.8291	0.001839296
Rnf144a	ring finger protein 144A	0.004320437	0.8288	0.001839296
Abca1	ATP-binding cassette, sub-family A (ABC1), member 1	0.000794327	0.8281	0.001839296
Coro1b	coronin, actin binding protein 1B	0.008979903	0.8278	0.001839296
LOC1005026(	hypothetical LOC100502669	0.008979903	0.827	0.001839296
Ppp1r3b	protein phosphatase 1, regulatory (inhibitor) subunit 3B	0.004320437	0.8269	0.001839296
Hnrnpa3	heterogeneous nuclear ribonucleoprotein A3	0.001933622	0.8267	0.001839296
Zfp697	zinc finger protein 697	0.000794327	0.8265	0.001839296
Sema6d	sema domain, transmembrane domain (TM), and cytoplasmic domain, (semaphorin) 6D	0.004320437	0.8256	0.001839296
Sepx1	selenoprotein X 1	0.001933622	0.8256	0.001839296
0610010F05R	RIKEN cDNA 0610010F05 gene	0.004320437	0.825	0.001839296
Sdccag3	serologically defined colon cancer antigen 3	0.001933622	0.8248	0.001839296
Wbscr27	Williams Beuren syndrome chromosome region 27 (human)	0.008979903	0.8248	0.001839296
Marcks	myristoylated alanine rich protein kinase C substrate	0.000794327	0.824	0.001839296
Aacs	acetoacetyl-CoA synthetase	0.001933622	0.8238	0.001839296
Csnk2b	casein kinase 2, beta polypeptide	0.004320437	0.8233	0.001839296
Kbtbd2	kelch repeat and BTB (POZ) domain containing 2	0.004320437	0.8224	0.001839296
Frmd4b	FERM domain containing 4B	0.008979903	0.8224	0.001839296
Sulf2	sulfatase 2	0.008979903	0.8219	0.001839296
Tmcc1	transmembrane and coiled coil domains 1	0.001933622	0.8218	0.001839296
D9Wsu74e	DNA segment, Chr 9, Wayne State University 74, expressed	0.004320437	0.8217	0.001839296
St3gal1	ST3 beta-galactoside alpha-2,3-sialyltransferase 1	0.008979903	0.8207	0.001839296
Mpdz	multiple PDZ domain protein	0.000794327	0.8203	0.001839296
Hnrpdl	heterogeneous nuclear ribonucleoprotein D-like	0.004320437	0.8199	0.001839296
Fmo1	flavin containing monooxygenase 1	0.008979903	0.8199	0.001839296
Polr3h	polymerase (RNA) III (DNA directed) polypeptide H	0.008979903	0.8186	0.002001342
Bcl9l	B-cell CLL/lymphoma 9-like	0.004320437	0.8185	0.002001342
Ccdc80	coiled-coil domain containing 80	0.004320437	0.8179	0.002001342
Rg9mtd2	RNA (guanine-9-) methyltransferase domain containing 2	0.001933622	0.8172	0.002001342
Endog	endonuclease G	0.001933622	0.8168	0.002001342
Arl5a	ADP-ribosylation factor-like 5A	0.004320437	0.8166	0.002001342
Fam110c	family with sequence similarity 110, member C	0.008979903	0.8149	0.002001342
Slc43a3	solute carrier family 43, member 3	0.008979903	0.8146	0.002001342
Ankrd9	ankyrin repeat domain 9	0.008979903	0.8144	0.002001342
Thra	thyroid hormone receptor alpha	0.008979903	0.8142	0.002001342
Rims4	regulating synaptic membrane exocytosis 4	0.004320437	0.8139	0.002001342
Rpp30	ribonuclease P/MRP 30 subunit (human)	0.004320437	0.8138	0.002001342
Nrarp	Notch-regulated ankyrin repeat protein	0.004320437	0.8138	0.002001342
Eif4ebp1	eukaryotic translation initiation factor 4E binding protein 1	0.001933622	0.8135	0.002001342
6430548M08R	RIKEN cDNA 6430548M08 gene	0.00665017	0.8134	0.002001342
Csnk1e	casein kinase 1, epsilon	0.000794327	0.8134	0.002001342
1600014C10R	RIKEN cDNA 1600014C10 gene	0.004320437	0.8132	0.002001342
Slc46a3	solute carrier family 46, member 3	0.008979903	0.8131	0.002001342
Abcc1	ATP-binding cassette, sub-family C (CFTR/MRP), member 1	0.008979903	0.8116	0.002001342
Mettl7a1	methyltransferase like 7A1	0.004320437	0.8116	0.002001342
Polr3k	polymerase (RNA) III (DNA directed) polypeptide K	0.008979903	0.8114	0.002001342
Apcdd1	adenomatosis polyposis coli down-regulated 1	0.008979903	0.8095	0.002169619
Ccrn4l	CCR4 carbon catabolite repression 4-like (S. cerevisiae)	0.004320437	0.809	0.002169619
Ttk	Ttk protein kinase	0.001933622	0.808	0.002169619
Hsp90aa1	heat shock protein 90, alpha (cytosolic), class A member 1	0.004320437	0.808	0.002169619
Adamts4	a disintegrin-like and metallopeptidase (reprolysin type) with thrombospondin type 1 mot	0.000294162	0.8077	0.002169619
Polr1d	polymerase (RNA) I polypeptide D	0.008979903	0.8075	0.002169619
Iqsec1	IQ motif and Sec7 domain 1	0.004320437	0.8074	0.002169619
Fam126b	family with sequence similarity 126, member B	0.001933622	0.8073	0.002169619
Dnajc10	DnaJ (Hsp40) homolog, subfamily C, member 10	0.004320437	0.8073	0.002169619
Tdg	thymine DNA glycosylase	0.004320437	0.8069	0.002169619
Mterfd3	MTERF domain containing 3	0.008979903	0.8067	0.002169619
Man2a2	mannosidase 2, alpha 2	0.008979903	0.8065	0.002169619
Khdrbs1	KH domain containing, RNA binding, signal transduction associated 1	0.001933622	0.8065	0.002169619

2310035K24R	RIKEN cDNA 2310035K24 gene	0.008979903	0.8065	0.002169619
Trak1	trafficking protein, kinesin binding 1	0.000794327	0.8057	0.002169619
Sra1	steroid receptor RNA activator 1	0.004320437	0.8048	0.002169619
Zfp598	zinc finger protein 598	0.008979903	0.8046	0.002169619
Rcbtb2	regulator of chromosome condensation (RCC1) and BTB (POZ) domain containing prote	0.000794327	0.8036	0.002308248
Nudt5	nudix (nucleoside diphosphate linked moiety X)-type motif 5	0.001933622	0.8035	0.002308248
Iws1	IWS1 homolog (S. cerevisiae)	0.001933622	0.8033	0.002308248
Akt3	thymoma viral proto-oncogene 3	0.008979903	0.8033	0.002308248
Abhd13	abhydrolase domain containing 13	0.001933622	0.8033	0.002308248
Pdgfb	platelet derived growth factor, B polypeptide	0.001933622	0.8031	0.002308248
Gabra3	gamma-aminobutyric acid (GABA) A receptor, subunit alpha 3	0.008979903	0.803	0.002308248
Klf9	Kruppel-like factor 9	0.000794327	0.8022	0.002308248
Nit1	nitrilase 1	0.000794327	0.8019	0.002308248
Fam76b	family with sequence similarity 76, member B	0.000794327	0.8014	0.002308248
Pmepa1	prostate transmembrane protein, androgen induced 1	0.004320437	0.8011	0.002308248
Tsc22d3	TSC22 domain family, member 3	0.004320437	0.8006	0.002308248
Zbtb38	zinc finger and BTB domain containing 38	0.000794327	0.8003	0.002308248
Ankrd54	ankyrin repeat domain 54	0.004320437	0.8	0.002308248
Car12	carbonic anyhydrase 12	0.008979903	0.8	0.002308248
Atp6v0c	ATPase, H+ transporting, lysosomal V0 subunit C	0.008979903	0.7992	0.002308248
Adss	adenylosuccinate synthetase, non muscle	0.008979903	0.7991	0.002308248
Wdfy3	WD repeat and FYVE domain containing 3	0.004320437	0.799	0.002308248
Car3	carbonic anhydrase 3	0.004320437	0.7974	0.002424431
Stau2	staufen (RNA binding protein) homolog 2 (Drosophila)	0.008979903	0.7973	0.002424431
Dynlt3	dynein light chain Tctex-type 3	9.57E-05	0.7972	0.002424431
Bnc2	basonuclin 2	0.001933622	0.7968	0.002424431
Nhlrc1	NHL repeat containing 1	0.000294162	0.7967	0.002424431
Abcb10	ATP-binding cassette, sub-family B (MDR/TAP), member 10	0.001933622	0.7964	0.002424431
Zcchc24	zinc finger, CCHC domain containing 24	0.008979903	0.7961	0.002424431
Ang	angiogenin, ribonuclease, RNase A family, 5	0.001933622	0.7956	0.002424431
Prr5l	proline rich 5 like	0.00665017	0.7948	0.002424431
Syvn1	synovial apoptosis inhibitor 1, synoviolin	0.001933622	0.7943	0.002424431
Mbnl2	muscleblind-like 2	0.000794327	0.7941	0.002424431
Dync1i2	dynein cytoplasmic 1 intermediate chain 2	0.004320437	0.794	0.002424431
Bmp1	bone morphogenetic protein 1	0.001933622	0.7939	0.002424431
Slc7a5	solute carrier family 7 (cationic amino acid transporter, y+ system), member 5	0.008979903	0.7929	0.002424431
Xrn1	5'-3' exoribonuclease 1	0.004320437	0.7922	0.002424431
Zfp703	zinc finger protein 703	0.000294162	0.7922	0.002523212
Rit1	Ras-like without CAAX 1	0.004320437	0.7922	0.002523212
Rcor3	REST corepressor 3	0.008979903	0.7919	0.002523212
Stxbp5l	syntaxin binding protein 5-like	0.000794327	0.7914	0.002523212
Poldip3	polymerase (DNA-directed), delta interacting protein 3	0.008979903	0.7913	0.002523212
Ube2h	ubiquitin-conjugating enzyme E2H	0.000794327	0.791	0.002523212
Lrrc59	leucine rich repeat containing 59	0.004320437	0.7904	0.002523212
Tmem168	transmembrane protein 168	0.008979903	0.79	0.002523212
LOC640502 //	UDP-N-acetylhexosamine pyrophosphorylase-like /// UDP-N-acetylglucosamine pyropho	0.004320437	0.7899	0.002523212
Vps13b	vacuolar protein sorting 13B (yeast)	0.008979903	0.7896	0.002523212
Bnip3	BCL2/adenovirus E1B interacting protein 3	0.004320437	0.7896	0.002523212
Ccl6	chemokine (C-C motif) ligand 6	0.004320437	0.7894	0.002523212
Aste1	asteroid homolog 1 (Drosophila)	0.008979903	0.789	0.002523212
Pbrm1	polybromo 1	0.008979903	0.789	0.002523212
Tusc5	tumor suppressor candidate 5	0.001933622	0.7887	0.002523212
Slc1a3	solute carrier family 1 (glial high affinity glutamate transporter), member 3	0.004320437	0.7884	0.002523212
Snrpd3	small nuclear ribonucleoprotein D3	0.008979903	0.7884	0.002523212
Syn2	synapsin II	0.008979903	0.7876	0.002523212
Abcb1a	ATP-binding cassette, sub-family B (MDR/TAP), member 1A	0.000794327	0.7869	0.002626268
Dsg2	desmoglein 2	0.008979903	0.7863	0.002626268
Fam107a	family with sequence similarity 107, member A	0.004320437	0.786	0.002626268
Snw1	SNW domain containing 1	0.001933622	0.7857	0.002626268
Gpatch1	G patch domain containing 1	0.008979903	0.7856	0.002626268
Ewsr1	Ewing sarcoma breakpoint region 1	0.001933622	0.7852	0.002626268
Anp32a	acidic (leucine-rich) nuclear phosphoprotein 32 family, member A	0.004320437	0.7851	0.002626268
Ntsr2	neurotensin receptor 2	0.001933622	0.785	0.002626268
Cdc42ep1	CDC42 effector protein (Rho GTPase binding) 1	0.004320437	0.7848	0.002626268
Hmgcs1	3-hydroxy-3-methylglutaryl-Coenzyme A synthase 1	0.008979903	0.7841	0.002626268
Ssfa2	sperm specific antigen 2	0.008979903	0.784	0.002626268
Bcar3	breast cancer anti-estrogen resistance 3	0.000794327	0.7831	0.002626268
Nanp	N-acetylneuraminic acid phosphatase	0.008979903	0.7829	0.002626268
Pfkp	phosphofructokinase, platelet	0.001933622	0.7825	0.002626268
Mgll	monoglyceride lipase	0.008979903	0.7818	0.00274943
Zeb2	zinc finger E-box binding homeobox 2	9.57E-05	0.7812	0.00274943
Ttc38	tetratricopeptide repeat domain 38	0.008979903	0.7799	0.00274943
Asxl1	additional sex combs like 1 (Drosophila)	0.004320437	0.7798	0.00274943
ENSMUSG00	predicted gene, ENSMUSG00000068790 /// alpha-takusan pseudogene /// predicted gene	0.008979903	0.7798	0.00274943
Clmn	calmin	0.008979903	0.7776	0.002861249
Lonrf3	LON peptidase N-terminal domain and ring finger 3	0.004320437	0.777	0.002861249
H3f3b	H3 histone, family 3B	0.000294162	0.777	0.002861249
Bmp4	bone morphogenetic protein 4	0.008979903	0.7764	0.002861249
Tob2	transducer of ERBB2, 2	0.008979903	0.7764	0.002861249
LOC1005033	hypothetical LOC100503380 /// matrin 3	0.008979903	0.7761	0.002861249

AI428301	expressed sequence AI428301	0.008979903	0.7759	0.002861249
Crym	crystallin, mu	0.000794327	0.7758	0.002861249
Ankrd13c	ankyrin repeat domain 13c	0.004320437	0.7752	0.002861249
Hnrnph3	heterogeneous nuclear ribonucleoprotein H3	0.001933622	0.7751	0.002861249
Ebf1	early B-cell factor 1	0.001933622	0.7744	0.002861249
5730403B10R	RIKEN cDNA 5730403B10 gene	0.008979903	0.7741	0.002861249
Nab2	Ngfi-A binding protein 2	0.008979903	0.7738	0.002861249
Nr1d1	nuclear receptor subfamily 1, group D, member 1	0.001933622	0.7726	0.002963223
Map3k11	mitogen-activated protein kinase kinase kinase 11	0.000794327	0.7725	0.002963223
Sepsecs	Sep (O-phosphoserine) tRNA:Sec (selenocysteine) tRNA synthase	0.008979903	0.7725	0.002963223
Fus	fusion, derived from t(12;16) malignant liposarcoma (human)	0.008979903	0.7722	0.002963223
Scyl3	SCY1-like 3 (S. cerevisiae)	0.000794327	0.7716	0.002963223
Plekhg1	pleckstrin homology domain containing, family G (with RhoGef domain) member 1	0.004320437	0.7714	0.002963223
Fam184a	family with sequence similarity 184, member A	0.004320437	0.7711	0.002963223
Ifrd2	interferon-related developmental regulator 2	0.004320437	0.7708	0.002963223
Ercc4	excision repair cross-complementing rodent repair deficiency, complementation group 4	0.004320437	0.7703	0.002963223
Glul	glutamate-ammonia ligase (glutamine synthetase)	0.000294162	0.77	0.002963223
Pik3ca	phosphatidylinositol 3-kinase, catalytic, alpha polypeptide	0.008979903	0.7699	0.002963223
Adrb3	adrenergic receptor, beta 3	0.008979903	0.7698	0.002963223
Tomm20	translocase of outer mitochondrial membrane 20 homolog (yeast)	0.008979903	0.7689	0.003065999
Sipa1l2	signal-induced proliferation-associated 1 like 2	0.004320437	0.7678	0.003065999
Rgs3	regulator of G-protein signaling 3	0.008979903	0.7678	0.003065999
Asph	aspartate-beta-hydroxylase	0.004320437	0.7675	0.003065999
Rasa3	RAS p21 protein activator 3	0.001933622	0.7662	0.003065999
Nr2f2	nuclear receptor subfamily 2, group F, member 2	0.008979903	0.7656	0.003161017
Pak3	p21 protein (Cdc42/Rac)-activated kinase 3	0.008979903	0.7654	0.003161017
9030624G23R	RIKEN cDNA 9030624G23 gene /// predicted gene 4425 /// predicted gene 5784 /// predi	0.008979903	0.7652	0.003161017
1810059H22R	RIKEN cDNA 1810059H22 gene	0.008979903	0.7651	0.003161017
Rhbdd3	rhomboid domain containing 3	0.004320437	0.7645	0.003161017
4933429F08R	RIKEN cDNA 4933429F08 gene	0.008979903	0.7642	0.003161017
Hdhd3	haloacid dehalogenase-like hydrolase domain containing 3	0.001933622	0.7639	0.003161017
Cdc14b	CDC14 cell division cycle 14 homolog B (S. cerevisiae)	0.008979903	0.7638	0.003161017
Ankrd11	ankyrin repeat domain 11	0.004320437	0.763	0.003161017
Taf9	TAF9 RNA polymerase II, TATA box binding protein (TBP)-associated factor	0.004320437	0.762	0.003249123
Stard5	StAR-related lipid transfer (START) domain containing 5	0.004320437	0.7619	0.003249123
Dmwd	dystrophia myotonica-containing WD repeat motif	0.004320437	0.7592	0.003331046
Arl2bp	ADP-ribosylation factor-like 2 binding protein	0.008979903	0.7591	0.003331046
Tmod2	tropomodulin 2	0.008979903	0.7589	0.003331046
Pfkfb3	6-phosphofructo-2-kinase/fructose-2,6-biphosphatase 3	0.000294162	0.7581	0.003331046
Zfp110	zinc finger protein 110	0.008979903	0.7581	0.003331046
Zc3h8	zinc finger CCCH type containing 8	0.004320437	0.7577	0.003331046
Spsb1	splA/ryanodine receptor domain and SOCS box containing 1	0.008979903	0.7576	0.003331046
Spsb1	splA/ryanodine receptor domain and SOCS box containing 1	0.008979903	0.7576	0.003331046
Rnps1	ribonucleic acid binding protein S1	0.008979903	0.7564	0.003331046
Itsn1	intersectin 1 (SH3 domain protein 1A)	0.008979903	0.7563	0.003331046
Tubb5	tubulin, beta 5	0.008979903	0.7548	0.003407414
AW209491	expressed sequence AW209491	0.004320437	0.7531	0.003407414
Lin7a	lin-7 homolog A (C. elegans)	0.008979903	0.753	0.003407414
2700078E11R	RIKEN cDNA 2700078E11 gene	0.008979903	0.7525	0.003478773
Arsk	arylsulfatase K	0.008979903	0.7512	0.003478773
Fam33a	family with sequence similarity 33, member A	0.008979903	0.7503	0.003478773
Dlg1	discs, large homolog 1 (Drosophila)	0.004320437	0.75	0.003545601
Sh3glb1	SH3-domain GRB2-like B1 (endophilin)	0.008979903	0.75	0.003545601
Gm10393 /// P	predicted gene 10393 /// placenta specific 9	0.000294162	0.7497	0.003545601
1190007F08R	RIKEN cDNA 1190007F08 gene	0.001933622	0.7484	0.003545601
Bzw2	basic leucine zipper and W2 domains 2	0.001933622	0.7484	0.003545601
Ipo7	importin 7	0.004320437	0.7483	0.003545601
Mr1	major histocompatibility complex, class I-related	0.008979903	0.7482	0.003545601
Wasf2	WAS protein family, member 2	0.004320437	0.7481	0.003545601
2310008H09R	RIKEN cDNA 2310008H09 gene	0.004320437	0.7473	0.003608316
Sp1	trans-acting transcription factor 1	0.001933622	0.747	0.003608316
Cyb5b	cytochrome b5 type B	0.008979903	0.7468	0.003608316
Trim16	tripartite motif-containing 16	0.008979903	0.7461	0.003608316
Smad4	MAD homolog 4 (Drosophila)	0.008979903	0.7455	0.003608316
Zranb2	zinc finger, RAN-binding domain containing 2	0.008979903	0.7454	0.003608316
Zbtb4	zinc finger and BTB domain containing 4	0.008979903	0.7454	0.003608316
Serbp1	serpine1 mRNA binding protein 1	0.008979903	0.7453	0.003608316
Rchy1	ring finger and CHY zinc finger domain containing 1	0.004320437	0.7453	0.003608316
Acsl1	acyl-CoA synthetase long-chain family member 1	0.000794327	0.7449	0.003608316
Ncaph2	non-SMC condensin II complex, subunit H2	0.004320437	0.7449	0.003608316
Med27	mediator complex subunit 27	0.004320437	0.7445	0.003667287
Flywch1	FLYWCH-type zinc finger 1	0.004320437	0.7443	0.003667287
Hspa12a	heat shock protein 12A	0.008979903	0.7436	0.003667287
Flot1	flotillin 1	0.004320437	0.7435	0.003667287
9530010C24R	RIKEN cDNA 9530010C24 gene	0.001933622	0.7435	0.003667287
Dhtkd1	dehydrogenase E1 and transketolase domain containing 1	0.001933622	0.7429	0.003667287
Glt25d1	glycosyltransferase 25 domain containing 1	0.000794327	0.7424	0.003667287
Plce1	phospholipase C, epsilon 1	0.000294162	0.7423	0.003667287
2210018M11R	RIKEN cDNA 2210018M11 gene	0.008979903	0.741	0.003722838
Nup54	nucleoporin 54	0.001933622	0.74	0.003722838

Mapk14	mitogen-activated protein kinase 14	0.008979903	0.74	0.003722838
Ndufb8	NADH dehydrogenase (ubiquinone) 1 beta subcomplex 8	0.008979903	0.7399	0.003722838
Tcf7l2	transcription factor 7-like 2, T-cell specific, HMG-box	0.008979903	0.7399	0.003722838
Pcyox1	prenylcysteine oxidase 1	0.004320437	0.7398	0.003722838
Il17rd	interleukin 17 receptor D	0.004320437	0.7397	0.003722838
Angptl2	angiopoietin-like 2	0.001933622	0.7388	0.003777719
Rxra	retinoid X receptor alpha	0.000794327	0.7379	0.003777719
Vps33a	Vacuolar protein sorting 33A (yeast)	0.000794327	0.7377	0.003777719
Rfwd2	ring finger and WD repeat domain 2	0.000794327	0.7367	0.003829658
Rorc	RAR-related orphan receptor gamma	0.008979903	0.7361	0.003829658
Ppp2r5a	protein phosphatase 2, regulatory subunit B (B56), alpha isoform	0.008979903	0.7354	0.003829658
Zbtb20	zinc finger and BTB domain containing 20	0.001933622	0.7353	0.003829658
Jarid2	jumonji, AT rich interactive domain 2	0.004320437	0.734	0.003878886
Shroom4	shroom family member 4	0.001933622	0.7336	0.003878886
Pbld	phenazine biosynthesis-like protein domain containing	0.008979903	0.7329	0.003878886
Serpine1	serine (or cysteine) peptidase inhibitor, clade E, member 1	0.008979903	0.7324	0.003925609
Nid2	nidogen 2	0.008979903	0.7321	0.003925609
Cox4nb	COX4 neighbor	0.008979903	0.7319	0.003925609
Tshz2	teashirt zinc finger family member 2	0.004320437	0.7316	0.003925609
Etv5	ets variant gene 5	0.008979903	0.7313	0.003925609
Pola1	polymerase (DNA directed), alpha 1	0.008979903	0.7313	0.003925609
Zc3h4	zinc finger CCCH-type containing 4	0.008979903	0.731	0.003925609
Lipe	lipase, hormone sensitive	0.004320437	0.7302	0.003970014
Tab3	TGF-beta activated kinase 1/MAP3K7 binding protein 3	0.008979903	0.7296	0.003970014
Gpt	glutamic pyruvic transaminase, soluble	0.008979903	0.7296	0.003970014
Morc4	microrchidia 4	0.008979903	0.7291	0.003970014
Fam102b	family with sequence similarity 102, member B	0.004320437	0.7283	0.003970014
Ablim2	actin-binding LIM protein 2	0.008979903	0.728	0.00401227
Fyn	Fyn proto-oncogene	0.004320437	0.728	0.00401227
Plin4	perilipin 4	0.000794327	0.728	0.00401227
Grb10	growth factor receptor bound protein 10	0.008979903	0.7275	0.00401227
Agpat9	1-acylglycerol-3-phosphate O-acyltransferase 9	0.004320437	0.7271	0.00401227
Wee1	WEE 1 homolog 1 (S. pombe)	0.004320437	0.7264	0.00401227
Pla2g16	phospholipase A2, group XVI	0.008979903	0.7262	0.00401227
Hspa8	heat shock protein 8	0.004320437	0.726	0.004052528
Nnat	neuronatin	0.008979903	0.7251	0.004052528
Rap2c	RAP2C, member of RAS oncogene family	0.004320437	0.7246	0.004052528
Ifi44	interferon-induced protein 44	0.000794327	0.7245	0.004052528
Zbtb46	zinc finger and BTB domain containing 46	0.008979903	0.724	0.004090927
Ppfia1	protein tyrosine phosphatase, receptor type, f polypeptide (PTPRF), interacting protein (li	0.00665017	0.7239	0.004090927
Wdr26	WD repeat domain 26	0.008979903	0.7237	0.004090927
Kdm6b	KDM1 lysine (K)-specific demethylase 6B	0.004320437	0.7224	0.004090927
Bcr	breakpoint cluster region	0.004320437	0.7212	0.004135621
Ppp1r2	protein phosphatase 1, regulatory (inhibitor) subunit 2	0.000294162	0.7209	0.004135621
Trip10	thyroid hormone receptor interactor 10	0.008979903	0.7208	0.004135621
Acot4	acyl-CoA thioesterase 4	0.000294162	0.7198	0.004178506
Fndc3a	fibronectin type III domain containing 3A	0.008979903	0.7193	0.004178506
Nudcd1	NudC domain containing 1	0.008979903	0.7192	0.004178506
Mdp1	magnesium-dependent phosphatase 1	0.008979903	0.7192	0.004178506
Rnd3	Rho family GTPase 3	0.001933622	0.7191	0.004178506
Ncbp2	nuclear cap binding protein subunit 2	0.008979903	0.7189	0.004178506
Med22	mediator complex subunit 22	0.001933622	0.7189	0.004178506
Igsf8	immunoglobulin superfamily, member 8	0.004320437	0.7183	0.004178506
Slc29a3	solute carrier family 29 (nucleoside transporters), member 3	0.001933622	0.718	0.004219688
B230214O09R	RIKEN cDNA B230214O09 gene	0.008979903	0.7174	0.004219688
Spred1	sprouty protein with EVH-1 domain 1, related sequence	0.004320437	0.7161	0.004259267
Pgpep1	pyroglutamyl-peptidase I	0.008979903	0.7161	0.004259267
Tmed10	transmembrane emp24-like trafficking protein 10 (yeast)	0.008979903	0.716	0.004259267
Dennd4a	DENN/MADD domain containing 4A	0.008979903	0.7158	0.004259267
Ptprd	protein tyrosine phosphatase, receptor type, D	0.001933622	0.7154	0.004259267
Tinagl1	tubulointerstitial nephritis antigen-like 1	0.004320437	0.7154	0.004259267
Ttc19	tetratricopeptide repeat domain 19	0.008979903	0.7153	0.004259267
1700021C14R	RIKEN cDNA 1700021C14 gene	0.004320437	0.7147	0.004259267
Frrs1	ferric-chelate reductase 1	0.008979903	0.7142	0.004300233
Pde4b	phosphodiesterase 4B, cAMP specific	0.000794327	0.7138	0.004300233
Fnbp1	formin binding protein 1	0.008979903	0.713	0.004300233
Pparg	peroxisome proliferator activated receptor gamma	0.008979903	0.7126	0.004339716
Trmt1	TRM1 tRNA methyltransferase 1 homolog (S. cerevisiae)	0.008979903	0.7119	0.004339716
Pebp1	phosphatidylethanolamine binding protein 1	0.004320437	0.7118	0.004339716
Ffar2	free fatty acid receptor 2	0.004320437	0.7115	0.004339716
Prelp	proline arginine-rich end leucine-rich repeat	0.008979903	0.7112	0.004339716
Arih2	ariadne homolog 2 (Drosophila)	0.008979903	0.7111	0.004339716
BC026513	cDNA sequence BC026513	0.004320437	0.7111	0.004339716
Klf13	Kruppel-like factor 13	0.008979903	0.711	0.004378313
Morf4l1	mortality factor 4 like 1	0.001933622	0.7101	0.004378313
Bcl7b	B-cell CLL/lymphoma 7B	0.008979903	0.7094	0.004378313
Agpat4	1-acylglycerol-3-phosphate O-acyltransferase 4 (lysophosphatidic acid acyltransferase, de	0.004320437	0.7092	0.004415571
Mrpl11	mitochondrial ribosomal protein L11	0.008979903	0.7092	0.004415571
Zfp422	zinc finger protein 422	0.008979903	0.7069	0.004451559
Chic1	cysteine-rich hydrophobic domain 1	0.008979903	0.7069	0.004451559

Gls	glutaminase	0.001933622	0.7068	0.004451559
Stam2	signal transducing adaptor molecule (SH3 domain and ITAM motif) 2	0.008979903	0.7067	0.004451559
Fam123b	family with sequence similarity 123, member B	0.004320437	0.7065	0.004451559
Gtf2i	general transcription factor II I	0.008979903	0.7064	0.004451559
Cdca4	cell division cycle associated 4	0.004320437	0.7056	0.004486339
Cask	calcium/calmodulin-dependent serine protein kinase (MAGUK family)	0.008979903	0.7045	0.004486339
Lrrc8d	leucine rich repeat containing 8D	0.004320437	0.7041	0.004519972
Srpk2	serine/arginine-rich protein specific kinase 2	0.008979903	0.704	0.004519972
Tacc1	transforming, acidic coiled-coil containing protein 1	0.004320437	0.7039	0.004519972
Snrk	SNF related kinase	0.008979903	0.703	0.004519972
A530020G20F	RIKEN cDNA A530020G20 gene	0.001933622	0.7027	0.004552514
Osbpl6	oxysterol binding protein-like 6	0.004320437	0.7021	0.004552514
Tor1b	torsin family 1, member B	0.008979903	0.702	0.004552514
Wrn	Werner syndrome homolog (human)	0.008979903	0.7	0.004584017
Dtx3l	deltex 3-like (Drosophila)	0.001933622	0.6999	0.004584017
Spg21	spastic paraplegia 21 homolog (human)	0.008979903	0.6998	0.004584017
Chic2	cysteine-rich hydrophobic domain 2	0.008979903	0.6991	0.00461453
Usp7	ubiquitin specific peptidase 7	0.008979903	0.6985	0.00461453
Ciao1	cytosolic iron-sulfur protein assembly 1 homolog (S. cerevisiae)	0.008979903	0.698	0.004644099
Fam19a5	family with sequence similarity 19, member A5	0.004320437	0.6979	0.004644099
Tspan4	tetraspanin 4	0.001933622	0.6966	0.004672766
Zdhhc3	zinc finger, DHHC domain containing 3	0.008979903	0.6966	0.004672766
Syap1	synapse associated protein 1	0.008979903	0.6962	0.004672766
5830407P18R	RIKEN cDNA 5830407P18 gene	0.001933622	0.696	0.004672766
Chmp4b	chromatin modifying protein 4B	0.008979903	0.6958	0.004672766
Tex264	testis expressed gene 264	0.008979903	0.6952	0.004700574
Iqgap2	IQ motif containing GTPase activating protein 2	0.004320437	0.6951	0.004700574
2610005L07R	cadherin 11 pseudogene	0.008979903	0.6949	0.004700574
Nck1	non-catalytic region of tyrosine kinase adaptor protein 1	0.008979903	0.6949	0.004700574
Ptplad1	protein tyrosine phosphatase-like A domain containing 1	0.008979903	0.6948	0.004700574
Fry	furry homolog (Drosophila)	0.004320437	0.6946	0.004700574
Dkc1	dyskeratosis congenita 1, dyskerin homolog (human)	0.008979903	0.6946	0.004700574
Egfl7	EGF-like domain 7	0.008979903	0.694	0.004733964
9130017K11R	RIKEN cDNA 9130017K11 gene	0.008979903	0.6933	0.004733964
4930447F24R	RIKEN cDNA 4930447F24 gene	0.004320437	0.6929	0.004733964
Cpsf6	cleavage and polyadenylation specific factor 6	0.004320437	0.6922	0.004766468
Mll5	myeloid/lymphoid or mixed-lineage leukemia 5	0.008979903	0.692	0.004766468
Fam171a1	family with sequence similarity 171, member A1	0.008979903	0.6899	0.00484317
Pank3	pantothenate kinase 3	0.004320437	0.6899	0.00484317
D7Wsu130e	DNA segment, Chr 7, Wayne State University 130, expressed	0.001933622	0.6895	0.00484317
Cbx5	chromobox homolog 5 (Drosophila HP1a)	0.004320437	0.6879	0.004880214
1700025G04R	RIKEN cDNA 1700025G04 gene	0.001933622	0.6877	0.004880214
Ppapdc2	phosphatidic acid phosphatase type 2 domain containing 2	0.000794327	0.6877	0.004880214
Dand5	DAN domain family, member 5	0.004320437	0.6871	0.004916425
Pdrg1	p53 and DNA damage regulated 1	0.008979903	0.6864	0.004916425
Gtf2h1	general transcription factor II H, polypeptide 1	0.008979903	0.6857	0.004951831
Apc	adenomatosis polyposis coli	0.008979903	0.6842	0.004986459
Siah1a	seven in absentia 1A	0.000794327	0.6839	0.004986459
Fscn1	fascin homolog 1, actin bundling protein (Strongylocentrotus purpuratus)	0.008979903	0.6835	0.004986459
Baz1b	bromodomain adjacent to zinc finger domain, 1B	0.008979903	0.6833	0.005020335
A030001D16F	RIKEN cDNA A030001D16 gene	0.008979903	0.683	0.005020335
Tbc1d5	TBC1 domain family, member 5	0.000794327	0.6817	0.005053482
Fbn1	fibrillin 1	0.008979903	0.6814	0.005053482
Kif1b	kinesin family member 1B	0.008979903	0.681	0.005053482
Eral1	Era (G-protein)-like 1 (E. coli)	0.008979903	0.681	0.005053482
Rpl15	ribosomal protein L15	0.004320437	0.6806	0.005085923
Anks1	ankyrin repeat and SAM domain containing 1	0.001933622	0.6798	0.005085923
Fdft1	farnesyl diphosphate farnesyl transferase 1	0.008979903	0.6798	0.005085923
Wdr35	WD repeat domain 35	0.004320437	0.6797	0.005117682
Epn2	epsin 2	0.000794327	0.6794	0.005117682
Nudt6	nudix (nucleoside diphosphate linked moiety X)-type motif 6	0.001933622	0.6788	0.005117682
Prdx6	peroxiredoxin 6	0.004320437	0.6761	0.005210644
Epb4.1l1	erythrocyte protein band 4.1-like 1	0.004320437	0.6757	0.005210644
5530400N10R	RIKEN cDNA 5530400N10 gene	0.008979903	0.6754	0.005210644
3632451O06R	RIKEN cDNA 3632451O06 gene	0.008979903	0.6739	0.005269549
Ddx5	DEAD (Asp-Glu-Ala-Asp) box polypeptide 5	0.004320437	0.6739	0.005269549
Tank	TRAF family member-associated Nf-kappa B activator	0.004320437	0.6738	0.005269549
1110037F02Ri	RIKEN cDNA 1110037F02 gene	0.008979903	0.6727	0.005298134
Il1r2	interleukin 1 receptor, type II	0.004320437	0.6727	0.005298134
Ints12	integrator complex subunit 12	0.008979903	0.6707	0.005326165
Map3k6	mitogen-activated protein kinase kinase kinase 6	0.004320437	0.6704	0.005353656
Sh2b2	SH2B adaptor protein 2	0.008979903	0.6697	0.005353656
Prnp	prion protein	0.001933622	0.6694	0.005380623
AI848100	expressed sequence AI848100	0.008979903	0.6693	0.005380623
Dact1	dapper homolog 1, antagonist of beta-catenin (xenopus)	0.008979903	0.6687	0.005380623
Rprd1b	regulation of nuclear pre-mRNA domain containing 1B	0.008979903	0.667	0.005433045
Hsph1	heat shock 105kDa/110kDa protein 1	0.008979903	0.667	0.005433045
Rev1	REV1 homolog (S. cerevisiae)	0.001933622	0.666	0.005458527
Zfp362	zinc finger protein 362	0.008979903	0.6658	0.005458527
Adrm1	adhesion regulating molecule 1	0.004320437	0.6649	0.005483541

Cd2bp2	CD2 antigen (cytoplasmic tail) binding protein 2	0.000294162	0.6643	0.005508101
Sdk2	sidekick homolog 2 (chicken)	0.008979903	0.6641	0.005508101
Srsf6	serine/arginine-rich splicing factor 6	0.008979903	0.6628	0.005532218
Nrg4	neuregulin 4	0.008979903	0.6628	0.005532218
Gabpb1	GA repeat binding protein, beta 1	0.008979903	0.6607	0.00557917
Ivns1abp	influenza virus NS1A binding protein	0.008979903	0.6604	0.00557917
Wscd2	WSC domain containing 2	0.004320437	0.6584	0.005646562
Gm129	predicted gene 129	0.008979903	0.6583	0.005646562
Il1f10	interleukin 1 family, member 10	0.008979903	0.6573	0.005668257
2610029I01Ri	RIKEN cDNA 2610029I01 gene	0.008979903	0.6568	0.005668257
Brd2	bromodomain containing 2	0.008979903	0.6566	0.005695348
Col9a1	collagen, type IX, alpha 1	0.001933622	0.6559	0.005695348
Csnk1d	casein kinase 1, delta	0.008979903	0.6556	0.005722037
Dtx4	deltex 4 homolog (Drosophila)	0.004320437	0.6548	0.005722037
Atp1a2	ATPase, Na+/K+ transporting, alpha 2 polypeptide	0.008979903	0.6548	0.005722037
Ube2i	ubiquitin-conjugating enzyme E2I	0.008979903	0.654	0.005748333
Xrcc3	X-ray repair complementing defective repair in Chinese hamster cells 3	0.008979903	0.6536	0.005774244
Cd59a	CD59a antigen	0.008979903	0.6532	0.005774244
Scarb2	scavenger receptor class B, member 2	0.001933622	0.6526	0.00579978
Grtp1	GH regulated TBC protein 1	0.008979903	0.6521	0.00579978
Pofut2	protein O-fucosyltransferase 2	0.008979903	0.652	0.005824947
Nop2	NOP2 nucleolar protein homolog (yeast)	0.000794327	0.6517	0.005824947
Fam101b	family with sequence similarity 101, member B	0.000794327	0.6506	0.005849755
Gtf2h2	general transcription factor II H, polypeptide 2	0.008979903	0.6503	0.005849755
Rhebl1	Ras homolog enriched in brain like 1	0.004320437	0.6499	0.005874209
2310004I24Ri	RIKEN cDNA 2310004I24 gene	0.008979903	0.6487	0.00589832
D18Ertd232e	DNA segment, Chr 18, ERATO Doi 232, expressed	0.004320437	0.6467	0.005968652
Dnajb2	DnaJ (Hsp40) homolog, subfamily B, member 2	0.004320437	0.6465	0.005968652
Tpd52	tumor protein D52	0.008979903	0.6462	0.005968652
Mobkl3	MOB1, Mps One Binder kinase activator-like 3 (yeast)	0.004320437	0.6453	0.005991452
Leo1	Leo1, Paf1/RNA polymerase II complex component, homolog (S. cerevisiae)	0.008979903	0.6427	0.006058016
Gata6	GATA binding protein 6	0.008979903	0.6419	0.006079611
Slc12a5	solute carrier family 12, member 5	0.008979903	0.6415	0.00610092
Prmt3	protein arginine N-methyltransferase 3	0.008979903	0.6342	0.006283725
Dbp	D site albumin promoter binding protein	9.57E-05	0.6317	0.006368093
Odf2	outer dense fiber of sperm tails 2	0.004320437	0.6308	0.006388588
Gdap10	ganglioside-induced differentiation-associated-protein 10	0.004320437	0.6303	0.006408852
Mcf2l	mcf.2 transforming sequence-like	0.004320437	0.63	0.006408852

Supplementary Table 2. Common rhythmic genes in mammary gland, cartilage and tendon tissues.

These 28 genes fall under the GO terms of "rhythmic process/regulators of transcription" (e.g. B*mal1, Clock, Per2/3, Cry1, Dbp, Tef, Hlf, Nr1d1/2, Tsc22d3* and *Leo1*), and "nuclear hormone receptors" (*Nr1d1/2* and *Thra*).

Adamts4
Arhgap20
Arntl
Cgrrf1
Clock
Cry1
D7Wsu130e
Dapk1
Dbp
Eef2k
Fmo1
Gm129
Hlf
Leo1
Nr1d1
Nr1d2
Per2
Per3
Plekhg1
Prr5l
Serpine1
Stard5
Tef
Thra
Tmem57
Tsc22d3
Vps13a
Wee1

Supplementary Table 3. List of antibodies used in this article.

ANTIBODY	SOURCE	IF	IB
Alexa Fluor 647 phalloidin	Thermo Fisher Scientific #A22287	1;100	
Bmal1 (mouse monoclonal)	Reference 9	1;1000	
Cy™5 AffiniPure Goat Anti-Rabbit IgG (H+L)	Jackson Immunoresearch #111-175-144	1;500	
ERK 2 Antibody (C-14)	Santa Cruz Biotechnology #sc-154		1;2000
Goat anti-Mouse IgG (H+L) Secondary Antibody, Alexa Fluor 488	Thermo Fisher Scientific #A-11001	1;500	
Human/Bovine/Mouse Integrin alpha 6/CD49f MAb (Clone GoH3)	R&D Systems #MAB13501	1;250	
Monoclonal Anti-α-Tubulin antibody produced in mouse	Sigma-Aldrich #T9026		1;2000
Monoclonal Anti-Vinculin antibody produced in mouse	Sigma-Aldrich #V4505	1;200	1;500
Mouse Monoclonal CD44 Antibody (8E2F3)	Novus Biologicals #NBP1-47386	1;250	
PER2 (rabbit polyclonal)	Reference 39	1;250	
Phospho-Myosin Light Chain 2 (Ser19) Mouse mAb	Cell Signaling Technology #3675	1;200	1;1000
Rho A Antibody (119)	Santa Cruz Biotechnology #sc-179		1;500
